# Generalized enzymatic mechanism of catalysis by tetrameric l-asparaginases from mesophilic bacteria

**DOI:** 10.1038/s41598-020-74480-4

**Published:** 2020-10-15

**Authors:** Pawel Strzelczyk, Di Zhang, Marzena Dyba, Alexander Wlodawer, Jacek Lubkowski

**Affiliations:** 1 Macromolecular Crystallography Laboratory, National Cancer Institute, Frederick, MD USA; 2 Basic Science Program, Structural Biophysics Laboratory, Frederick National Laboratory for Cancer Research Sponsored by the National Cancer Institute, Frederick, MD USA

**Keywords:** Enzyme mechanisms, X-ray crystallography

## Abstract

The mechanism of catalysis by the l-glutaminase-asparaginase from *Pseudomonas 7A* (PGA) was investigated using structural, mass spectrometry, and kinetic data. We had previously proposed mechanism of hydrolysis of l-Asn by the type II l-asparaginase from *E. coli* (EcAII), but that work was limited to just one enzyme. Based on results presented in this report, we postulate that all homotetrameric l-asparaginases from mesophilic bacteria utilize a common ping-pong mechanism of catalysis consisting of two subsequent nucleophilic substitutions. Several new structures of non-covalent complexes of PGA with different substrates, as well as structures of covalent acyl-enzyme intermediates of PGA with canonical substrates (l-Asp and l-Glu) and an opportunistic ligand, a citrate anion, were determined. The results of kinetic experiments monitored by high-resolution LC/MS, when combined with new structural data, clearly show that the reaction catalyzed by l-glutaminase-asparaginases proceeds through formation of a covalent intermediate, as observed previously for EcAII. Additionally, by showing that the same mechanism applies to l-Asn and l-Gln, we postulate that it is common for all these structurally related enzymes.

## Introduction

l-asparaginases (EC 3.5.1.1), first mentioned by Clementi in 1922^1^ are widely distributed enzymes among both prokaryotic and eukaryotic organisms. Their primary biochemical function is to catalyze the hydrolysis of l-Asn to l-Asp. Most of l-asparaginases also catalyze the hydrolysis of l-Gln to l-Glu, and those with glutaminase activity comparable or higher than asparaginase activity are often referred to as glutaminases-asparaginases (EC 3.5.1.38). Two structurally related l-asparaginases are present in the model bacterium *Escherichia coli*, with their names abbreviated EcAI (for cytoplasmic enzyme) and EcAII (for periplasmic enzyme).Asparaginases from two bacterial sources (EcAII from *E. coli* and ErA from *Erwinia chrysanthemi* (*Dickeya dadantii*)) came to a focus in 1960s–1970s, after being identified as successful agents in treatment of various leukemias and lymphomas, prominently the juvenile non-Hodgkin, lymphoblastic leukemia (ALL)—the most prevalent pediatric cancer (reviewed in^2–4^). Effectiveness of l-asparaginases is based on a principle of amino acid restriction. Since some cancer cells (notably several lines of hemolytic cancers) have impaired capacity to synthesize l-asparagine, depletion of l-Asn leads to alteration of metabolic pathways, resulting in starvation and death^5^. However, l-Asn starvation may have a broader therapeutic potential, as recent reports indicate that l-asparaginase has shown significant inhibitory effect on metastasis of breast cancer^6^ and positive therapeutic effect in treatment of pancreatic cancer^7^. Due to their efficacy and to economic factors^5^, l-asparaginases remain the first-line drugs in the treatment of ALL, despite recent developments of immunotherapies^8,9^. Several modified preparations of these enzymes with reduced immunogenicity and increased stability, have been developed since addition of l-asparaginases to the therapeutic repertoire^2^. Nevertheless, despite all advances and a high rate of therapeutic success in ALL, several deficiencies of asparaginase therapy have been reported. A still debated question is whether substrate specificity, particularly the l-glutaminase activity, is required for clinical efficiency^10^, or it is mainly a contributor to side effects^11–13^.

Despite a long history of therapeutic uses of l-asparaginases, mechanistic details of biochemical reactions catalyzed by these enzymes had been ambiguous^14,15^. Recently, we described a plausible model for the mechanism of catalysis assisted by these enzymes^16^. Our previous studies focused exclusively on EcAII, an enzyme with low l-glutaminase activity, and left open a question whether conclusions drawn from that work apply to a broader range of l-asparaginases, particularly to enzymes with significant l-glutaminase activity.

### Pseudomonas 7A

l-glutaminase-asparaginase (PGA; EC 3.5.1.38) shares 48% amino acid sequence identity with EcAII, but was reported to hydrolyze l-Gln with nearly two-fold higher efficiency than l-Asn^17^. Only four structures of PGA had been deposited in the Protein Data Bank (PDB) before commencement of this work. They represent ligand-free PGA (PDB ID 3pga;^18^), PGA with the active site occupied by an opportunistic ligand SO_4_ (PDB ID 4pga)^19^, as well as two complexes of PGA with irreversible inhibitors, 4,4-dihydroxy-5-oxo- l-norvaline (DONV; PDB ID 1djo) and 5,5-dihydroxy-6-oxo- l-norleucine (DON; PDB ID 1djp)^20^. The latter two structures are of only limited utility in describing the catalytic process for canonical substrates, since the inhibitors bind covalently in a non-conventional way to both Thr20 and Tyr34, and do not mimic the standard catalytic process^20,21^. Notably, structures of PGA with natural substrates, l-Gln/ l-Glu and l-Asn/ l-Asp, have not yet been described.

Anticancer properties of PGA were investigated previously^22,23^ and this enzyme was subsequently found unsuitable for anticancer treatment due to its low proteolytic stability and significant side effects^24^. Like EcAII, PGA originates from mesophilic bacteria and its biological assembly is a homotetramer. The active sites of both enzymes are chemically and structurally nearly identical, despite of great differences of their substrate specificities. Detailed structural studies of PGA and its complexes should create a foundation for rational development of l-asparaginases with modulated relative activities vs. l-Asn or l-Gln^25^, which appears to be of major importance in developing improved anti-leukemia therapeutics.

## Results

### Catalytic activity of PGA preparations

Since in this study we focused solely on the catalytic process, i.e. on molecular events taking place during conversion of the Michaelis complex and the enzyme:product complex, our evaluation was limited to determination of appropriate turnover numbers (*k*_cat_ values) and did not include parameters related to the binding process (i.e. K_M_ values). Catalytically deficient variants of PGA were selected for this study by following strategies developed previously for EcAII^16^. To estimate the *k*_cat_ values of PGA(wt), PGA(T100V), and PGA(K173M), we performed a series of kinetic experiments (as described in Materials and Methods), in which we monitored formation of ammonia resulting from hydrolysis of l-Asn and l-Gln, or of NH_2_OH derived from l-AHA. We made sure that all reactions proceeded at the maximum rate (V_max_). In all cases concentrations of a substrate (l-Asn, l-Gln, or l-AHA) were much higher (> 140,000x) than of the enzyme concentration, and well above (> 1000) the reported respective K_M_ values. Furthermore, based on previous studies^16,26^ indicating that the only source of inhibition during these assays is due to reactions with products (l-Asp or l-Glu, but only under acidic conditions). To minimize product inhibition, we made sure that during the assay less than 10% of the substrate (l-Asn of l-Gln) would undergo hydrolysis. Thus, product inhibition effects were negligible^16^ and, if present, would be detectable from non-linear trend of the progress curves. The individual progress curves are shown in Supplementary Fig. S1a-d.

The estimated *k*_cat_ values for hydrolysis of l-Asn and l-Gln by PGA(wt) are 31+/− 3 s^−1^ and 36+/− 4 s^−1^, respectively; those values are significantly lower for the mutated variants, i.e. in the case of PGA(T100V), *k*_cat_(l-Gln) = 0.0072 s^−1^ and *k*_cat_(l-Asn) = 0.0061 s^−1^, while in the case of PGA(K173M), *k*_cat_(l-Gln) = 0.00033 s^−1^ and *k*_cat_(l-Asn) = 0.00028 s^−1^ (for comparison, *k*_cat_(l-AHA) = 0.00023 s^−1^). Based on the data published by Roberts^17^, the K_M_ values of PGA are comparable (4.4 μM for l-Asn and 4.6 μM for l-Gln)^17^. These authors also reported that PGA hydrolyzes l-Gln more efficiently than l-Asn^17^. However, PGA-catalyzed hydrolysis appears to be less efficient compared to EcAII (*k*_cat_ = 45–49 s^−1^)^27,28^. EcAII, however, catalyzes hydrolysis of l-Gln nearly two orders of magnitude less efficiently than l-Asn. Substitutions Thr100 → Val or Lys173 → Met render catalytically deficient PGA variants, similar to the previously reported data for EcAII^16^ (see also Supplementary Fig. S1b-d), whereas the double mutant PGA(T100V, K173M) does not have any measurable activity. We also observed that in the case of PGA either of the two substitutions reduce the catalytic activity significantly more than in the case of EcAII. The reasons for these differences are currently not clear.

### Studies of covalent intermediates in solution

In recent studies of EcAII we have shown that covalent complexes formed during co-crystallization represent intermediates of enzymatic reactions^16,29^. To validate this approach for PGA we performed mass spectrometry experiments, checking whether covalent complexes are formed within the time frame (i.e. 0.5–1 min) that could be explained only in terms of an enzymatic processes, and that molecular weights of these entities agree with those predicted for covalent acyl-enzyme intermediates (AEIs). The measurements were performed in a pH range 4—8, that allowed detection of AEIs and indicated a pH range optimal for subsequent crystallization studies. In our experiments we used l-Asp and l-Glu, which act as substrates in enzymatically-controlled oxygen exchange reactions, as described previously^26,30^:

$${\text{E}} + {\text{X}} - {\text{COO}}^{{\text{n}}} {\text{H }} + {\text{ H}}_{{2}} {\text{O}}^{{\text{m}}} \rightleftarrows {\text{E }} + {\text{ X}} - {\text{COO}}^{{\text{m}}} {\text{H }} + {\text{ H}}_{{2}} {\text{O}}^{{\text{n}}}$$

Assuming that this reaction proceeds through formation of covalent AEI, the equilibrium can be simplified:

$${\text{E}} + {\text{X}} - {\text{COOH}} \rightleftarrows {\text{AEI }} + {\text{ H}}_{{2}} {\text{O}}$$

**Figure 1:**
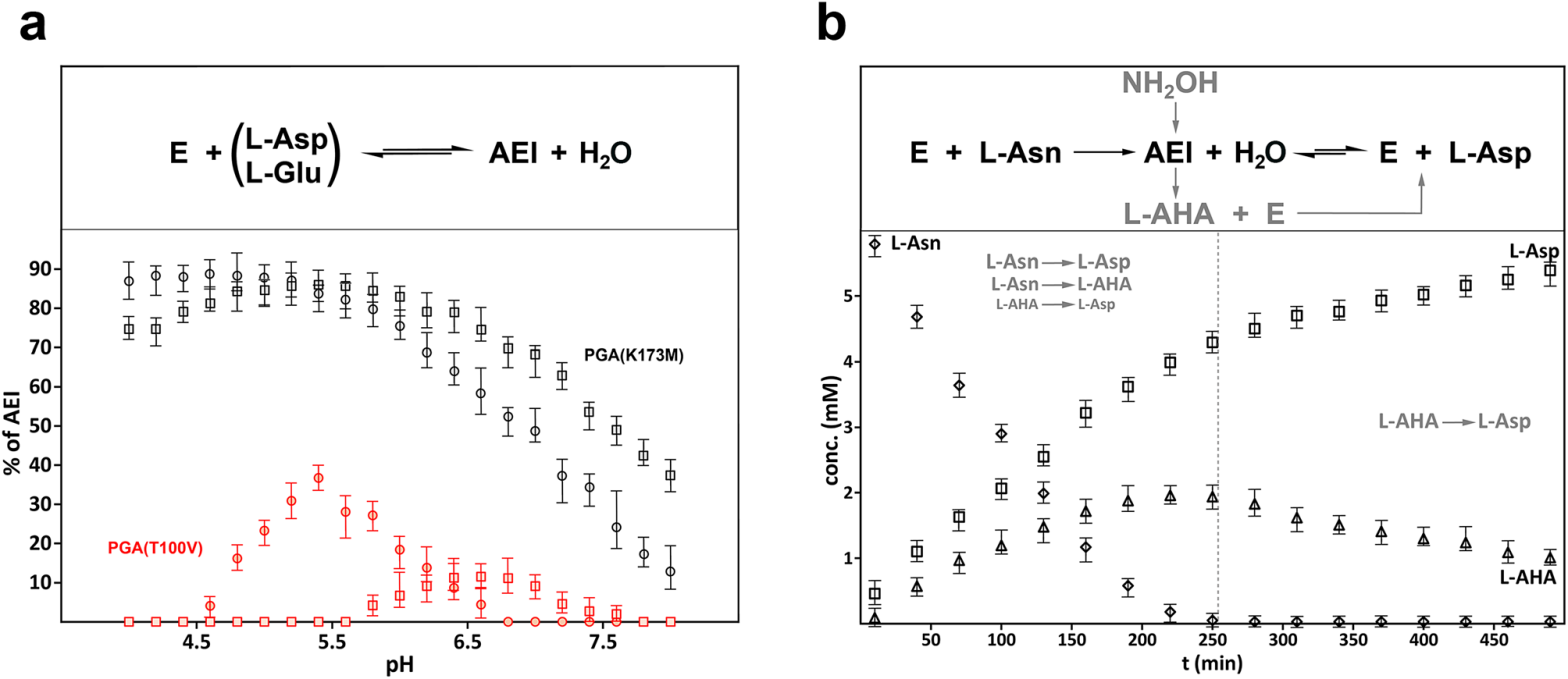
Mass Spectrometry studies of reactions between PGA and its substrates. Formation of covalent acyl-enzyme intermediates (AEI) in mixtures of L-aspartic (or L-glutamic) acid with either PGA(K173M) or PGA(T100V) at different pH values, measured by Single Quad LC/MS, is illustrated in panel (**a**). The equilibria present in each mixture are shown at the top of this panel. Resulting data points, obtained for solutions containing PGA(K173M) and PGA(T100V) are colored in black and red, respectively. Values recorded for experiments with L-Glu are marked by squares and those obtained in mixtures containing L-Asp by circles. Each data point represents an average from three independent measurements and the range of individual readings is represented by vertical error bars. Content of AEI (% AEI) was calculated according to the formula (% AEI = abundance of AEI [%])/{(abundance of AEI [%]) + (abundance of EcAII(K162M) [%])}. Progress of L-Asn hydrolysis by PGA(wt) in the presence of an external nucleophile (NH_2_OH) is shown in panel (**b**). At each time point, concentrations of substrate (L-Asn), product (L-Asp), and byproduct (L-AHA) were determined by Q-TOF LC/MS as described in Materials and Methods. Concentrations of L-Asn, L-Asp and L-AHA are marked by diamonds, squares, and triangles, respectively. Vertical dashed line indicates the time at which all L-Asn was converted to either L-Asp or L-AHA. The reactions taking place prior this time are listed on the left of the dashed line, in gray font. The irreversible reaction taking place in the absence of L-Asn is shown to the right of the dashed line. Relationship between reactions taking place during this experiment is illustrated at the top of this panel. Since this assay was conducted at pH 7.7, equilibrium between L-Asp and L-AHA is negligible (see panel **a**). Each data point represents an average from three independent measurements, with the ranges of individual readings represented by vertical error bars. Figure was prepared with the licensed programs Microsoft Excel and Adobe Photoshop CC 2019.

Covalent AEI can be detected if the rate of the forward reaction is faster compared to the reverse one. In such a case, AEI should be detectable by a number of different experimental methods. As we argued previously^16^, such equilibrium cannot be achieved with the native substrates, l-Asn or l-Gln, because the release of NH_3_ precludes reversibility of the reaction and the relative rates of acylation and deacylation prevent accumulation of covalent intermediates. Therefore, for all structural studies described here we used either l-Asp or l-Glu. In these experiments, mixtures of catalytically deficient mutants, PGA(K173M) or PGA(T100V), with l-Asp (or l-Glu) buffered to exact pH values, were subjected to LC/MS analysis. Results of these measurements are illustrated in Fig. 1a. The relative amounts of PGA(K173M) and AEI were assumed to be proportional to relative mass abundances reported by the software used for analysis of the Single-Quad LC/MS results. The experimental molecular weights of the enzyme and AEIs agree within 1 Da with the predicted values. Molar concentrations of both components were not determined.

As seen in Fig. 1a, AEIs are formed in a broad pH range in all four experiments (two variants and two substrates). Compared to PGA(T100V) variant, the relative content of AEI is significantly higher in the case of PGA(K173M). This suggests that for the latter variant the rate of deacylation is lower compared to the rate of acylation. While in the case of PGA(K173M) covalent intermediates are detected throughout a whole studied pH range, for the PGA(T100V) variant the pH ranges are significantly narrower. Differences reported here for the two variants result from the fact that the K173M substitution (in contrast to T100V) eliminates a charged active site residue, despite the fact that hydrophobicity of the active site pocket is increased in both cases. Based on these results, the PGA(K173M) variant appears to be more suitable for structural studies of covalent intermediates than PGA(T100V).

As indicated earlier, l-asparaginases catalyze the oxygen exchange reaction under slightly acidic conditions (promoting protonation of either l-Asp or l-Glu). Therefore, it might be expected that reaction rates should increase with a decrease of pH values and this effect should be reflected by an increase in the amounts of AEIs. Results presented in Fig. 1a show that, in the case of PGA(K173M), the maximum contents of AEIs are observed at pH 5.3 for l-Glu and 4.7 for l-Asp. These values are higher by ~ 1 pH unit than the standard p*K*_a_ values for both amino acids in water solutions^31^. They are even more elevated (by an additional pH unit) in the case of PGA(T100V). Structural analysis discussed later in this report indicates that an interaction between the side chains of productively-bound l-Asp or l-Glu and the main chain oxygen of Ser125 promotes protonation of carboxyl groups, leading to a decrease of their acidity. Introduction of a hydrophobic side chain (i.e. T100V substitution) in direct vicinity of the carboxylate of the substrate, further decreases the acidity of the latter. As explained previously^16^, a decrease of the AEI abundance, observed for both variants at low pH, is most likely associated with protonation of other charged groups, i.e. α-carboxylates of substrates.

### Detection of AEI in reactions with canonical substrates (l-Asn or l-Gln)

In our previous report^16^ we postulated that formation of AEIs is not detected when canonical substrates (l-Asn or l-Gln) are used, due to slow acylation followed by rapid deacylation. Such a conclusion, however, leads to a rather incomplete description of the enzymatic mechanism. To fill this gap, we devised experiments utilizing high-resolution Q-TOF LC/MS with canonical substrates, l-Asn or l-Gln. We based these experiments on the previously published strategy^30,32^ that incorporates the use of an external nucleophile to probe formation of AEI. In our experiments, the substrate (l-Asn or l-Gln) and an external nucleophile (NH_2_OH), were mixed with PGA(wt). The external nucleophile was expected to react with the transiently-formed AEI, replacing a water molecule involved in the second nucleophilic substitution. This would lead to formation of either l-AHA or l-GHA instead of l-Asp or l-Glu. Figure 1b provides a more detailed description of chemical reactions taking place during this experiment. Although this approach strongly suggests formation of covalent AEI^32^, the original authors considered a possibility of reaction between NH_2_OH and non-covalently bound substrate (l-Asn). Due to the utilization of a different technology our approach was greatly simplified and the results could now be interpreted together with the results of structural studies. First, we determined by high-resolution Q-TOF LC/MS the m/z values for the small-molecule reactants (l-Asp, l-Asn, l-Glu, l-Gln, l-AHA, l-GHA, HEPES buffer). Subsequently, we performed a series of time-lapsed measurements with mixtures containing PGA(wt), EcAII(wt), or EcAII(T12V), with a significant molar excess of l-Asn, l-Asp, or l-Gln at pH 7.7, in the presence of NH_2_OH. We also acquired mass spectrometry data for the mixtures of l-Asn or l-Gln with NH_2_OH over a long period of time. Finally, using calibration curves relating mass spectrometry response of ions to defined concentrations of l-Asn, l-Asp, l-Gln, l-Glu, l-AHA, and l-GHA, we were able to create profiles of concentrations for all the significant components.

We found that, in the absence of PGA (or EcAII used as a reference), l-Asn or l-Gln do not react with NH_2_OH under conditions of this assay. Similarly, no products were detected when l-Asp or l-Glu were mixed with the enzymes in the presence of NH_2_OH at pH 7.7. These observations are in agreement with the results presented in Fig. 1a and confirm that both l-Asp and l-Glu serve as substrates of l-asparaginase only when protonated. Concentration changes of l-Asn, l-Asp, and l-AHA for the reaction between l-Asn and PGA(wt) in the presence of NH_2_OH, are shown in Fig. 1b. It is quite clear that l-AHA is generated during reaction between the enzyme and l-Asn. When the original substrate l-Asn is exhausted, enzymatic hydrolysis of l-AHA to l-Asp still proceeds. Under conditions of the assay, the reverse reaction proceeds with a negligible rate and stops when both l-Asn and l-AHA are exhausted. Equivalent results were obtained when reactions between PGA(wt) and l-Gln or EcAII(wt) with l-Asn were monitored (not shown). While a clear advantage of the assays performed here over those reported previously^30,32^ is the ability to simultaneously monitor concentrations of the substrate, product, and byproduct, it is still not possible to discount a possibility of forming a byproduct without covalently engaging the enzyme.

A critical experiment involved mixtures of the EcAII(T12V) variant with l-Asn in the presence of NH_2_OH. No l-AHA was generated in such mixtures under conditions of the assay over a period of several hours. Structure of EcAII(T12V) with l-Asn was published by us previously^33^, and it demonstrated that binding of the substrate is identical to that observed for the wild-type EcAII. Therefore, if formation of a non-covalent complex would lead to some form of substrate activation, it should also be evident in the complex with the EcAII(T12V) variant. This series of experiments provides quite clear proof that hydrolysis of l-Asn (or l-Gln) catalyzed by l-asparaginases proceeds through formation of a covalent intermediate (AEI) and conforms to the ping-pong mechanism.

### Crystallographic studies

**Figure 2:**
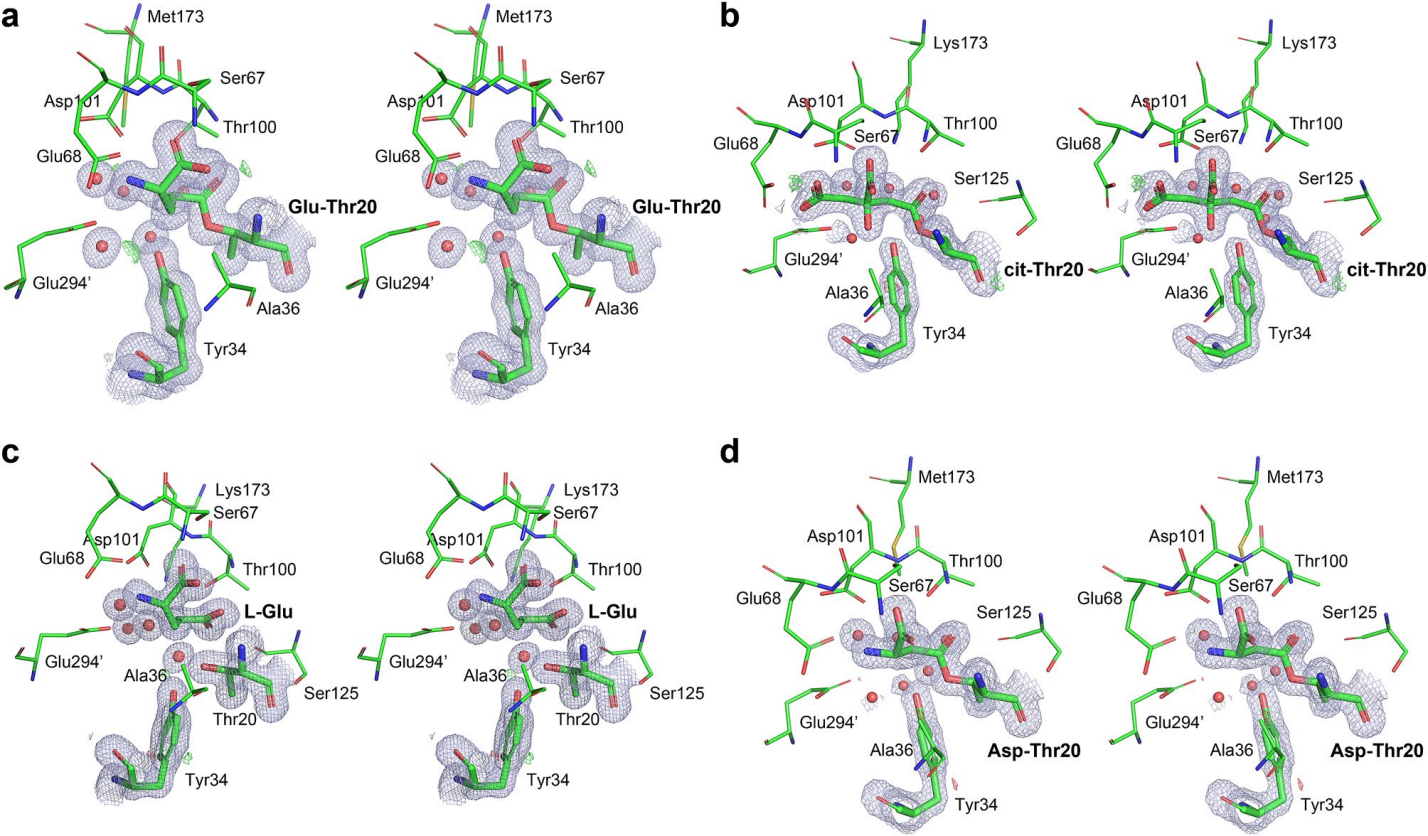
Representative electron densities of the ligands and parts of the active sites of PGA. For clarity, the 2mF_o_-DF_c_ maps, contoured at 1.0·σ level (blue) cover only the ligands, solvent, and the most labile components of the active site. No significant peaks are visible in the *m*F_o_-*D*F_c_ difference maps extending over the entire active site at the contour levels ± 3·σ. (**a**) Covalent acyl-enzyme intermediate L-Glu-Thr20, found in the active site of PGA(K173M) mutant (structure 6, resolution 1.15 Å), crystallized at pH 5. (**b**) Covalent acyl-enzyme intermediate, citrate-Thr20, found in the active site of PGA(wt) crystallized at pH 5.5 (structure 7, resolution 1.70 Å). (**c**) L-Glu bound non-covalently in the active site of PGA(wt) crystallized at pH 6.5 (structure 3, resolution 1.35 Å). (**d**) Covalent acyl-enzyme intermediate L-Asp-Thr20 found in the active site of the PGA(K173M) mutant (structure 5, resolution 1.58 Å) crystallized at pH 6. Figure was prepared with the licensed programs PyMol ver. 2.3.2, (Schrodinger LLC) and Adobe Photoshop CC 2019.

Seven new structures presented in this report were solved and refined using X-ray data collected at the Advanced Photon Source in Argonne National Laboratory from frozen single crystals. Crystals of non-covalent complexes and of covalent intermediates were obtained by co-crystallization of the wild-type PGA or the catalytically-deficient PGA(K173M) variant with appropriate ligands. Crystallization conditions are presented in Table 1. All structures, except structure 1, were refined at a very high resolution (Table 2), but even in the case of structure 1 the redundancy and consistency of X-ray data resulted in high quality electron density maps and allowed for the unambiguous interpretation and modeling. Four examples of electron density maps and modeled structures are shown in Fig. 2.

**Table 1:** Details of crystallization conditions.

Structure (crystal)	Crystallization conditions	Crystal composition
1	** Sample: PGA(wt)** 13.3 mg/ml, L-Asp 10 mM **Precipitant:** 0.1 M sodium acetate pH 4.5, 25%(w/v) PEG3350 **Cryo solution:** precipitant with 25%(v/v) glycerol and 5 mM L-Asp	**Non-covalent** complex with **L-Asp**
2	** Sample: PGA(wt)** 13.3 mg/ml, L-Asp 10 mM **Precipitant:** Tacsimate 8% (w/v) pH 5, 20%(w/v) PEG3350 **Cryo solution:** precipitant with 20%(v/v) glycerol and 5 mM L-Asp	**Non-covalent** complex with **L-Asp**
3	** Sample: PGA(wt)** 13 mg/ml, L-Glu 10 mM **Precipitant:** Tacsimate 8% (w/v) pH 6.5, 17%(w/v) PEG3350 **Cryo solution:** precipitant with 20%(v/v) ethylene glycol and 10 mM L-Glu	**Non-covalent** complex with **L-Glu**
4	** Sample: PGA(K173M)** 12.5 mg/ml, D-Glu 10 mM **Precipitant:** 0.1 M MgCl_2_, 50 mM Bis–Tris buffer pH 5.5, 20%(w/v) PEG3350 **Cryo solution:** precipitant with 20%(v/v) ethylene glycol and 10 mM D-Glu	**Non-covalent** complex with **D-Glu**
5	** Sample: PGA(K173M)** 12.6 mg/ml, L-Asp 10 mM **Precipitant:** Tacsimate 8% (w/v) pH 6, 20%(w/v) PEG3350 **Cryo solution:** precipitant with 20%(v/v) glycerol and 5 mM L-Asp	Acyl-enzyme **intermediate** with **L-Asp**
6	** Sample: PGA(K173M)** 13.3 mg/ml, L-Glu 10 mM **Precipitant:** 0.2 M sodium malonate pH 5, 20%(w/v) PEG3350 **Cryo solution:** precipitant with 20%(v/v) glycerol and 5 mM L-Glu	Acyl-enzyme **intermediate** with **L-Glu**
7	** Sample: PGA(wt)** 13.2 mg/ml, L-Glu 10 mM **Precipitant:** 0.1 M sodium citrate pH 5.5, 18%(w/v) PEG3350 **Cryo solution:** precipitant with 25%(v/v) glycerol	Acyl-enzyme **intermediate** with **citrate**

**Table 2:** Statistics of data collection and structure refinement.

Structure	1	2	3	4	5	6	7
Space group	*P*3_1_	*P*3_1_	*P*2_1_	*P*3_1_	*P*2_1_	*P*2_1_	*P*2_1_
Unit cell parameters (Å, *degrees*)	80.73, 80.73, 176.40	80.96, 80.96, 176.82	81.54, 130.79, 81.55, 117.8	81.28, 81.28, 176.70	78.03, 129.75, 81.41, 118.7	78.63, 130.46, 81.41, 119.0	74.15, 77.52, 137.13, 104.5
**Data collection Statistics**							
Completeness (%)	97.2 (48.4)	99.8 (98.3)	96.9 (94.5)	99.6 (98.8)	98.7 (96.4)	93.0 (53.9)	96.0 (67.9)
Redundancy	5.7 (5.1)	8.3 (4.9)	2.7 (2.6)	9.5 (6.4)	3.8 (3.3)	6.3 (3.2)	5.8 (3.7)
I/σ(I)	27.3 (5.6)	27.4 (2.2)	16.0 (2.0)	27.4 (1.9)	14.9 (2.1)	27.1 (2.3)	17.2 (2.6)
Unique reflections	71,715 (1789)	216,530 (10,676)	319,520 (15,558)	142,831 (7101)	191,717 (9296)	470,476 (13,625)	159,631 (5642)
High resolution shell	2.11 – 2.15	1.48 – 1.51	1.35 – 1.37	1.70 – 1.73	1.58 – 1.61	1.15 – 1.17	1.70 – 1.73
R-linear	0.061 (0.267)	0.076 (0.737)	0.048 (0.547)	0.088 (1.092)	0.088 (0.725)	0.060 (0.428)	0.121 (0.641)
R-square	0.058 (0.282)	0.070 (0.711)	0.036 (0.532)	0.067 (1.056)	0.068 (0.644)	0.047 (0.418)	0.096 (0.644)
R_pim_	0.028 (0.127)	0.027 (0.349)	0.033 (0.402)	0.030 (0.452)	0.051 (0.453)	0.025 (0.260)	0.054 (0.370)
CC_1/2_	0.995 (0.941)	0.996 (0.732)	0.973 (0.579)	0.995 (0.660)	0.990 (0.654)	0.997 (0.808)	0.992 (0.742)
**Refinement statistics**							
Resolution (Å)*	2.13–39.4	1.48–35.1	1.35–34.8	1.70–39.6	1.58–33.1	1.15–34.5	1.70–38.8
	(2.13–2.18)	(1.48–1.52)	(1.35–1.39)	(1.70–1.75)	(1.58–1.62)	(1.15–1.18)	(1.70–1.74)
**No. reflections**							
Refinement	68,787	2,04,714	3,02,518	1,37,133	1,86,557	4,44,163	1,56,067
Validation	2783	3013	3218	3728	3747	3771	2300
Protein chains in a.u.^@^	(A,B) & (C,D)	(A,B) & (C,D)	(A,B) & (C,D)	(A,B) & (C,D)	(A,B) & (C,D)	(A,B) & (C,D)	(A,B) & (C,D)
**No. of non-H atoms**							
Total	10,507	10,945	11,171	10,855	11,137	12,030	11,534
Water	870	1244	1157	1274	1125	1795	1485
Ligands	36	42	90	40	32	48	48
Type of ligand	Non-covalent complex with L-Asp	Non-covalent complex with L-Asp	Non-covalent complex with L-Glu	Non-covalent complex with D-Glu	Covalent AEI with L-Asp	Covalent AEI with L-Glu	Covalent AEI with citrate
**Conformation of **^a^							
HR^%^	A-D in *cat* +	A-D in *cat-*	C,D in *cat* +	A-D in *cat* +	A-D in *cat* +	A-D in *cat* +	A-D in *cat* +
ASFL	A-D in *cat-*	A-D in *cat-*	A,B in ~ *cat-*^b^	disordered A-D	A-D in *cat* +	A-D in *cat* +	A-D in *cat* +
ADP model^&^	Isotropic	Anisotropic	Anisotropic	Isotropic	Isotropic	Anisotropic	Isotropic
**Average ADP (Å2): total**							
Protein	27.7	17.3	16	19.6	16.7	20.4	17.8
Water	27.2	15.7	14.7	18.1	15.4	18	16
	33	29.9	26.2	31.1	28	34.4	29.8
R-factor	0.142 (0.175)	0.088 (0.161)	0.123 (0.244)	0.171 (0.284)	0.139 (0.236)	0.100 (0.426)	0.141 (0.216)
R_free_	0.198 (0.239)	0.120 (0.261)	0.149 (0.301)	0.215 (0.304)	0.157 (0.224)	0.120 (0.363)	0.166 (0.248)
**Rmsd values**							
Bond lengths (Å)	0.019	0.018	0.021	0.02	0.013	0.018	0.014
Bond angles (˚)	2.183	1.89	2.121	2.233	1.804	2.043	1.878
Est. coordinate error (Å)^#^	0.115	0.022	0.018	0.079	0.043	0.016	0.054
PDB ID	6wyw	6wyx	6wyy	6wyz	6wz4	6wz6	6wz8

*Numbers in parentheses refer to the high-resolution shell.

^&^ADP, atomic displacement parameters.

^#^Estimate based on maximum likelihood.

^@^In parentheses are monomers forming an intimate dimer.

^%^HR—hinge region, ASFL—active site flexible loop (see text for details).

^a^See text for definition of *cat* + and *cat-*

^b^assignment ~ *cat-* means that the main chain of HR follows *cat* + , however, the side chain of Thr20 assumes a non-catalytic orientation.

### Non-covalent complexes of PGA

In this report, we present structures of two non-covalent complexes between PGA(wt) and l-Asp (structures 1 and 2), one complex between PGA(wt) and l-Glu (structure 3), and the complex PGA(K173M):D-Glu (structure 4). While almost undistinguishable, structures 1 and 2 were determined using diffraction data collected from crystals grown under somewhat different conditions (see Table 1). In contrast to structure 1, where the isotropic model was used for refinement of atomic displacement parameters (ADPs), higher resolution of X-ray data allowed us to refine structure 2 with anisotropic ADPs.

**Figure 3:**
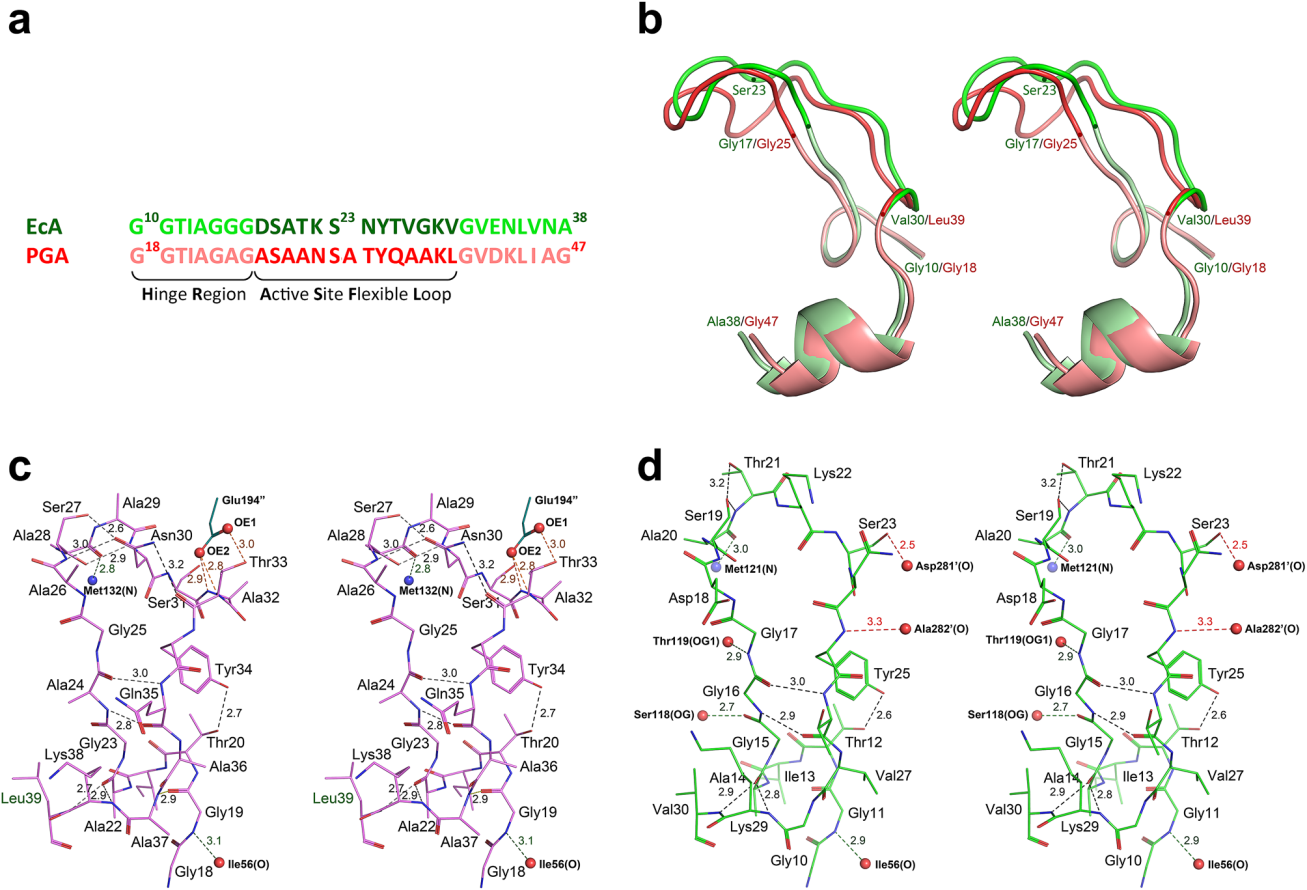
Catalytic (*cat*+) conformations of the HR and ASFL regions in PGA and EcAII. (**a**) Structure-based amino acid sequence alignment for the extended sections containing HR and ASFL motifs of both enzymes. (**b**) Ribbon representation of the aligned sections of PGA (protomer C in structure 3, complex with L-Glu, shades of red) and EcAII (protomer A in 6pac, complex with L-Asp, shades of green), corresponding to sequences shown in panel **a**. Differences in conformations of two ASFLs, resulting from one residue insertion in PGA, are clearly visible. **(c)** Structural details of HR and ASFL in the complex of PGA with L-Glu (monomer C in structure 3). A majority of H-bonds (colored black) are between atoms within flexible fragment shown in this panel, stabilizing its conformation. Two H-bonds, marked in green, are formed with atoms from other fragments of the same protomer (labeled with smaller bold font) and additional two H-bonds, shown in orange, with Glu194", contributed by a protomer from another dimer. (**d**) An equivalent fragment of the complex of EcAII with L-Asp (protomer A in 6pac). In panels **c** and **d,** all H-bonds are marked with dashed lines, assisted by corresponding distances. Figure was prepared with the licensed programs PyMol ver. 2.3.2, (Schrodinger LLC) and Adobe Photoshop CC 2019.

In both structures 1 and 2 part of the active site flexible loop (ASFL) (a.a. 26–35), including Tyr34 that is critical for catalysis, is disordered despite high resolution of diffraction data. Flexibility of the N-terminal region of l-asparaginases was identified during early crystallographic studies of these enzymes^18,34^. Two motifs, the “hinge region” (HR) and ASFL, were subsequently defined^16^. Both ASFL and HR play a critical role in catalysis by l-asparaginases^16^. The former region contributes the catalytically important Tyr34, whereas the latter one contains Thr20, the critical primary nucleophile. Figure 3a shows the location and alignment of both fragments in PGA and EcAII; their superposition is shown in Fig. 3b.

Notably, ASFL is one residue longer in PGA than in EcAII, resulting in a different conformation (Fig. 3c,d). This difference may contribute to distinctive substrate specificities of both enzymes, a problem that is not analyzed in this manuscript. For clarity of subsequent discussion, we re-emphasize here the previously introduced concept of productive (or catalytically-competent, *cat* +) and non-productive (*cat-*) state or conformation of ASFL and HR^16^. As explained earlier, ASFL assumes the unique *cat* + state only in the presence of a substrate and only when HR is also in *cat* + conformation, otherwise it remains in a disordered (*cat-*) state. Whereas the conformation of the HR region remains unchanged, its orientation relative to the enzyme active site changes from *cat-* in the absence of the substrate to *cat* + upon substrate binding. In the latter conformation, threonine residue (Thr20 in PGA or Thr12 in EcAII) is reoriented to the position suitable for nucleophilic attack.

**Figure 4:**
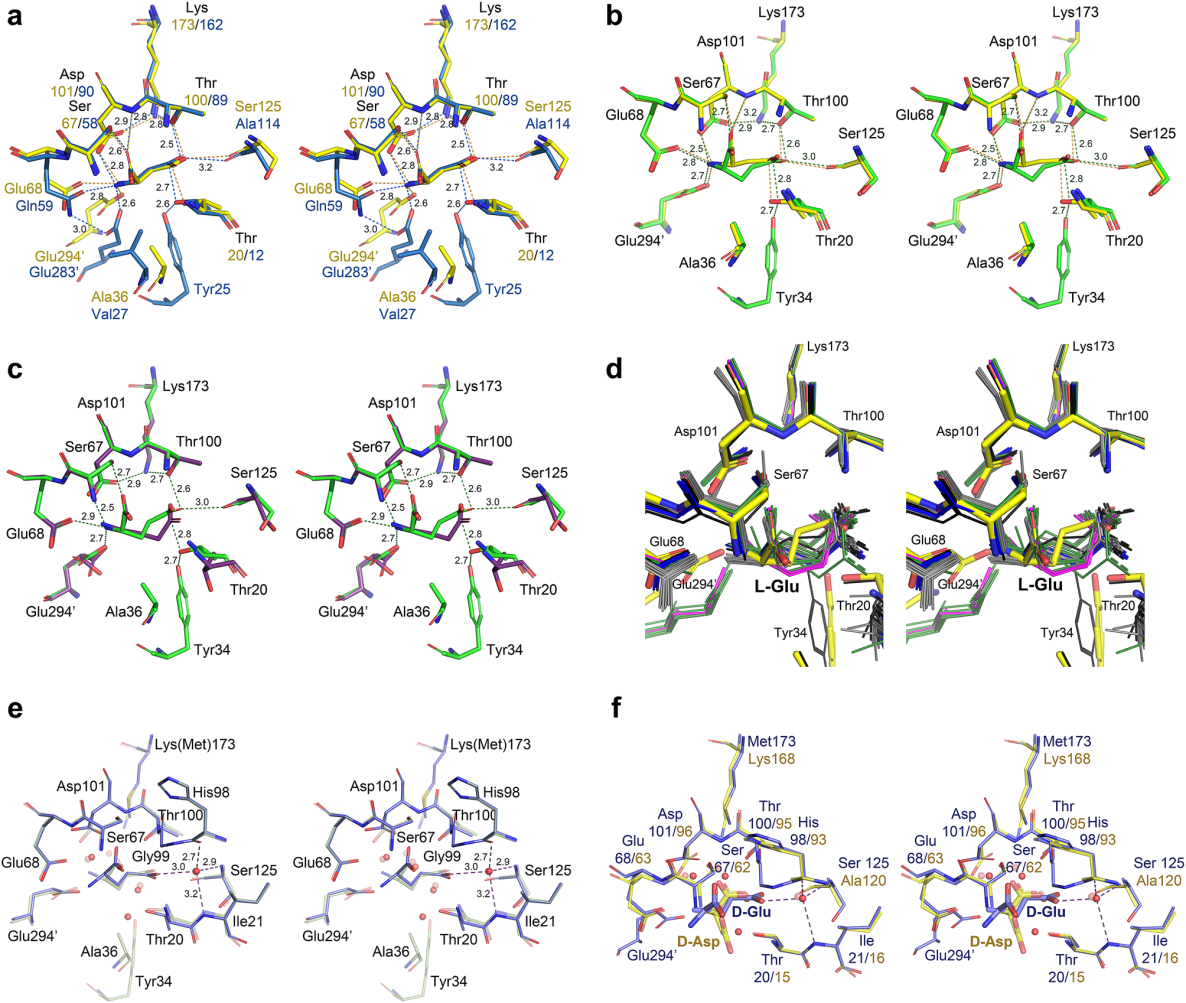
Complexes of PGA with L-Asp and L-Glu. (**a**) Superimposed active sites of PGA (yellow, protomer B in structure 2) and EcAII (blue, protomer A in structure 6pac) in complex with L-Asp. Equivalent residues are labeled. Labels with primed numbers indicate residues contributed by another protomer from the same tight dimer. Important interactions, common to both complexes, are indicated with dashed lines; however, for clarity the corresponding distances are shown for the EcAII:L-Asp complex only. Tyr34 was not visible in the PGA complex. (**b**) Comparison of L-Asp and L-Glu binding to the PGA active site. L-Asp complex (yellow) represents the active site B in structure 2 (Tyr34 not visible). Complex with L-Glu (green) represents the active site C in structure 3. Conserved H-bonds are indicated by dashed lines for both complexes; however, for clarity the distances are shown for the PGA:L-Glu complex only. (**c**) Two binding modes in PGA:L-Glu complexes. Superimposed active sites of PGA are shown for the complexes with L-Glu bound in *cat* + (green, protomer C in structure 3) and *cat-* (dark violet, protomer B in structure 3) conformations. Important interactions (primarily H-bonds) between the substrate and active site residues are indicated by dashed lines with corresponding distances. (**d**) Structures of L-asparaginase/L-Glu complexes compared to the complex in PGA. Aligned fragments of all active sites shown as thin sticks are colored green (5k4h), magenta (5k45), black (5hw0), gray (2hln), and blue (1hfw). Active site C from the PGA complex (structure 3) is shown in thick yellow sticks. (**e**) Structural alignment of two non-covalent complexes PGA(K173M):D-Glu (blue) and PGA(wt):L-Glu (semi-transparent gray). (**f**) Superposition of the active sites of PGA(K173M):D-Glu (blue) and ErA(wt):D-Asp (semi-transparent yellow). Figure was prepared with the licensed programs PyMol ver. 2.3.2, (Schrodinger LLC) and Adobe Photoshop CC 2019.

Structure of PGA in the complex with l-Asp is the first one reported for this ligand with any l-glutaminase-asparaginase. Superposition of the active site of PGA(wt): l-Asp complex (monomer B in structure 2) on the active site of EcAII(wt): l-Asp complex is shown in Fig. 4a. Structural similarity of both complexes and the relationship of substrate molecules to the catalytic residues (Thr20, Thr100, Asp101, and Lys173 in PGA) are striking. Positions of l-Asp molecules, as well as of the catalytic residues, align almost exactly. The largest structural deviation between the equivalent components of the two active sites is seen for PGA(Glu294′) and EcAII(Glu283′), residues contributed by another protomer within the biological assembly. A smaller discrepancy is seen between the positions of PGA(Ala36) and Val27_EcAII_. Three structural features are responsible for these differences. Both PGA(Glu294′)/EcAII(Glu283′) and PGA(Ala36)/EcAII(Val27) are contributed by hairpins interacting with each other, i.e. in the case of PGA hairpin consisting a.a. 288′–307′ and the ASFL. Compared to EcAII, a single-residue insertion in PGA is present in each of these hairpins, altering their conformation. An additional effect may be attributed to the substitution Glu → Gln at position 68 in PGA, the residue directly interacting with the side chain of PGA(Glu294′). Important H-bonded interactions in the active sites are indicated in Fig. 4a with dashed lines for both superimposed complexes. For clarity, distances are shown for only the EcAII: l-Asp complex. Equivalence of these H-bonds in both structures, however, is quite apparent. Among important interactions between the l-Asp with the active site residues that are shared by both enzymes, the H-bond between l-Asp(OD1) and the PGA(Ser125,O) atom, and the presentation of PGA(Thr20,OH) (the primary nucleophile) against l-Asp(CG), are quite noticeable. Both contacts are prerequisites for subsequent catalytic reaction and are the hallmark of productive substrate binding. In summary, the binding mode and presentation of l-Asp in the active site of PGA is nearly identical to that found in EcAII. As mentioned earlier, the relative positions of l-Asp(OD1) and PGA(Ser125,O) atoms, consistently observed for the *cat* + substrate binding leads to lowering the acidity of the l-Asp (or l-Glu) side chain carboxylate groups.

Binding of l-Glu, the other canonical substrate, to the active site of PGA(wt) is shown in Fig. 4b, depicting monomer C in structure 3 superimposed on the PGA(wt): l-Asp complex. In the case of this high-resolution structure, we observed two modes of binding of l-Glu. Productive (*cat* +) binding was seen in protomers C and D, and non-productive (*cat-*) binding in protomers A and B. Whereas complete protein chains could be modeled in protomers C and D, part of ASFL (including Tyr34) was disordered in protomers A and B.

Inspection of Fig. 4b, representing non-covalent complexes between PGA and l-Asp or l-Glu, leads to two significant observations. First, conformations and positions of the active site residues are identical in both structures. Secondly, despite differences in sizes of the side chains of l-Asp and l-Glu, their functional groups bound to PGA occupy identical sites, resulting in both complexes being involved in equivalent networks of H-bonds. Differences in sizes of the two amino acids are compensated by a particular conformation of the C_α_-C_β_ and C_β_-C_γ_ bonds in the l-Glu substrate, in which the C_β_ atom is projected towards ASFL, in particular in the direction of two residues, Thr20 and Ala36.

The two modes of binding l-Glu to the PGA active site are illustrated in Fig. 4c. In both structures the active site residues align very well, except of Tyr34 and Thr20 that are contributed by ASFL and HR, respectively. The latter two fragments are either disordered (ASFL) or accommodate *cat-* conformation (HR), which is intimately related to non-productive binding of l-Glu. While substrate molecules could be accommodated in the active site pocket in both cases, in the *cat-* conformation C_γ_-atoms of l-Glu protrude toward catalytic Thr20, preventing the latter from assuming the *cat* + conformation. Also, the side chain carboxylate in this complex is rotated relative to the catalytic complex and projects away from Thr20. As a result, the two interactions characteristic for the catalytic complex, with Ser125(O) and Thr20(OH), cannot be established.

It is important to note that structure 3 is not the first one for an l-asparaginase in complex with l-Glu. Previously, five structures of equivalent complexes were described for ErA^35–37^ and l-asparaginase from *Wolinella succinogenes* (WsA)^38^). In Fig. 4d we show a superposition of all 32 independent active sites of ErA and WsA occupied by l-Glu and the active site of PGA with l-Glu in *cat* + conformation. When comparing the ErA and WsA active sites with that of PGA, it is clear that structural differences are mirroring those described above for the PGA active sites with l-Glu bound in *cat-* and *cat* + conformations. In nearly all previously published ErA and WsA active sites ASFL is disordered and HR is in the *cat-* conformation. Also, it is quite apparent that in those structures l-Glu assumes a different conformation than the *cat* + observed in the active sites C and D of the PGA(wt): l-Glu complex.

Several experiments involving soaking of the ligand-free crystals of PGA in solutions containing l-Glu were performed as part of this study. In all cases (not shown here) we observed non-productive binding resulting in disordered ASFL, quite similar to the examples shown in Fig. 4c,d. Based on these results, we suggest that binding of l-Glu to PGA (and possibly to other l-asparaginases) proceeds as a two-step process (see “Discussion”). Significantly, the PGA(wt): l-Glu non-covalent complex shown here (Fig. 4c,d) represents productive binding of l-Glu to l-asparaginase. It is also important to notice that *cat* + binding modes of l-Asp and l-Glu are completely equivalent (Fig. 4b), an observation that is quite significant in the context of the catalytic mechanism.

Compared to EcAII, ErA, or WsA, PGA is more promiscuous towards its substrates^17^. It was reported that hydrolysis of l-Gln and D-Gln is catalyzed by PGA with higher efficiently than that of l-Asn, and hydrolysis of D-Asn reaches nearly 70% of that measured for l-Asn^17^. In order to investigate the binding and catalysis of the D-enantiomorphs of canonical substrates we determined the structure of a non-covalent complex PGA(K173M):D-Glu (structure 4). Superposition of this structure and the PGA(wt): l-Glu complex is shown in Fig. 4e. In the complex with D-Glu, ASFL (a.a. 23–39) is disordered and not modeled in any of the four protomers. Aside from that difference, the active site residues in both complexes assume nearly identical conformations and positioning of the side chain of D-Glu toward Ser125(O) and Thr20(OG) agrees with the expected *cat* + conformation. Furthermore, the α-carboxyl groups of both substrates occupy identical positions and make equivalent interactions with the enzyme. A major difference, resulting from different stereochemistry of the substrates, is location of the α-amino group. While in D-Glu this group points toward the side chain of Glu294′, forming a well-defined H-bond with this residue and with the side chain of Glu68, in l-Glu this group projects toward the side chain of Asp101, causing its rotation. In this complex the α-amino group of the substrate forms three well-defined H-bonds, with Asp101, Glu68 and Glu294'.

The only other previously reported structure of l-asparaginase in complex with a D-stereoisomer of a substrate was that of ErA:D-Asp (PDB ID 1hg1), refined at 1.8 Å resolution^35^. That structure was obtained by soaking crystals of presumably ligand-free enzyme in solution of D-Asp. Similarly to the PGA(K173M):D-Glu complex reported here, ASFLs are disordered in all four protomers in the ErA:D-Asp complex. Importantly, the side chain of D-Asp in that structure points in the direction of ASFL and HR, preventing both regions from assuming *cat* + conformations.

Superposition of the active sites from PGA(K173M):D-Glu and ErA:D-Asp complexes is shown in Fig. 4f. This figure clearly shows that D-Asp accommodates *cat-* conformation, suggesting that binding of this substrate to the ErA active site is not energetically favorable. Therefore, the structure of the PGA(K173M):D-Glu complex reported here is the first example of a catalytic complex with the D-stereoisomer of the substrate.

### Covalent complexes of PGA

Recently, we described a covalent AEI formed by EcAII and l-Asp and proved that its formation is a result of an enzymatic process^16^. This was the only example of a covalent complex between l-asparaginase and its canonical substrate. As discussed earlier, detection of AEI proves that catalytic reaction follows the double-displacement mechanism^16^. Earlier we have also shown that type I l-asparaginase from *E. coli* (EcAI) forms covalent AEI with a citrate anion, that is structurally equivalent to AEI with l-Asp^29^.

One of the main goals of the current work was to demonstrate that catalytic hydrolysis by a broader range of l-asparaginases shares the same mechanism. Here, we describe two structures of covalent AEIs formed by the PGA(K173M) variant and its canonical substrates, l-Asp (structure 5) and l-Glu (structure 6). We also describe a covalent complex of PGA(wt) with a citrate anion (structure 7). These structures have been determined and refined at high resolution and the resulting models are characterized by very good statistics (Table 2). The final electron density maps, shown for the active site region in Fig. 2a,b,d, clearly show formation of covalent bonds between the ligands and Thr20. Crystals of all three complexes belong to the same space group, *P*2_1_. Structures 5 and 6 are isomorphous, whereas different crystal packing is seen in structure 7. In all three structures the contents of asymmetric units represent the whole homo-tetrameric biological assemblies and HR and ASFL motifs accommodate *cat* + conformations.

**Figure 5:**
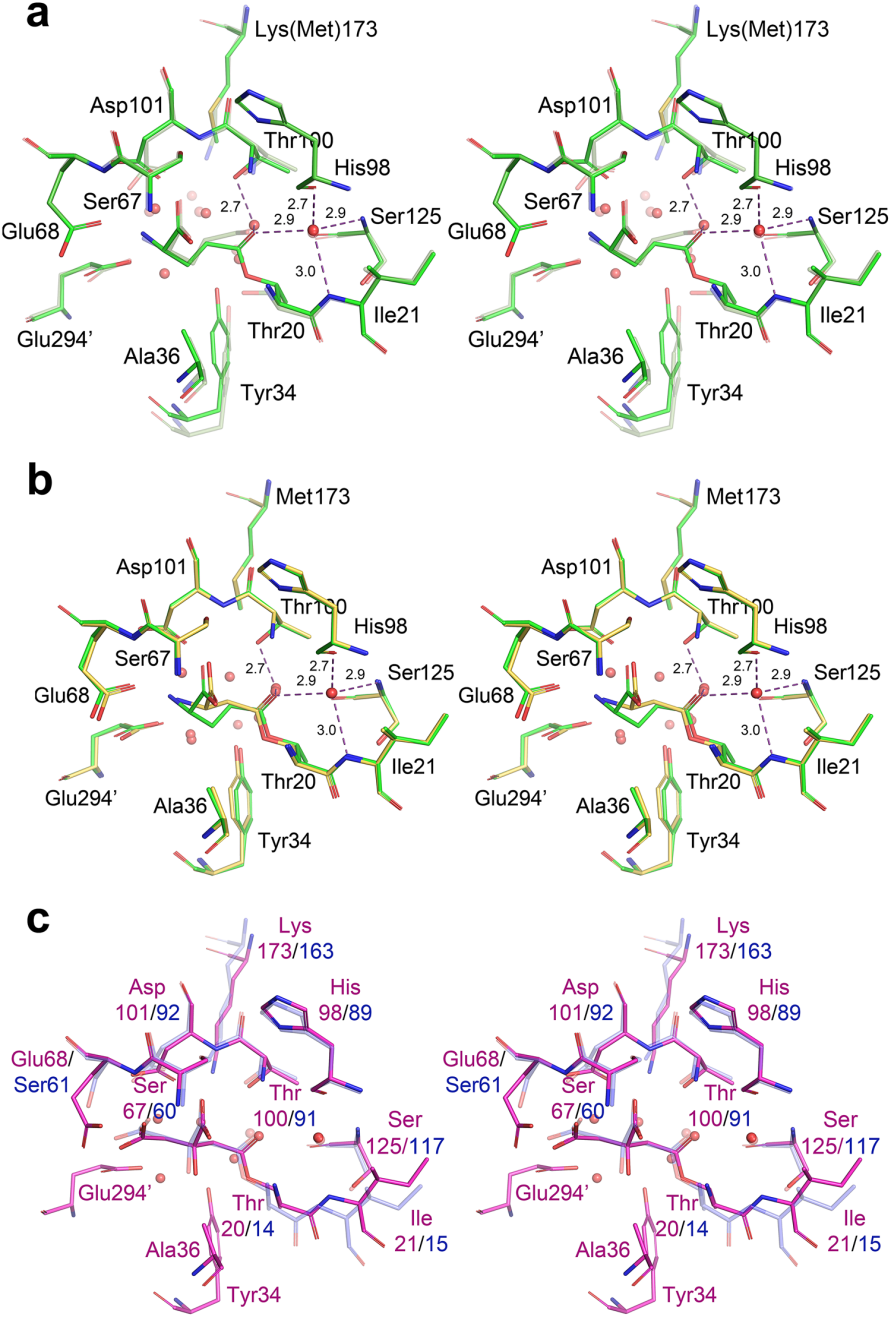
Active site of PGA with covalently-bound ligands and the non-covalent complex with L-glutamic acid. (**a**) Superposition of a covalent complex of PGA(K173M) and L-Glu (green) with non-covalent complex between PGA(wt) and L-Glu (semi-transparent gray). H-bonds engaging two important water molecules are indicated by dashed lines and the associated distances are shown. (**b**) Near-perfect structural equivalence of the covalent complexes of PGA(K173M) with L-Asp (yellow) or L-Glu (green). (**c**) Superposition of covalent complexes between PGA(wt) (magenta) and EcAI(wt) (6nxd) (semi-transparent blue) with citrate. Most of ASFL could not be modeled in the EcAI(wt) complex. Despite of low amino acid sequence conservation and different substrate specificities of the two enzymes, binding of citrate to their active sites is almost identical. Figure was prepared with the licensed programs PyMol ver. 2.3.2, (Schrodinger LLC) and Adobe Photoshop CC 2019.

By comparing structures of the non-covalent complex of PGA(wt) and l-Glu with that of the corresponding covalent AEI (Fig. 5a), it is apparent that structural changes within the active site pockets are minimal. These changes are limited to modest shifts of three side chains (of the substrate, Thr20, and Tyr34). An analogous observation was previously reported for the reaction between EcAII and l-Asp^16^. In fact, formation of a covalent bond between Thr20(O_γ_) and l-Glu(O_δ_) only marginally affects positions of the adjacent water molecules. A superposition of covalent AEI formed by the canonical substrates, illustrated in Fig. 5b, shows that transiently formed covalent bonds have identical stereochemistry. A discrepancy between positions of the C_β_ atoms of the superimposed substrates, also observed in equivalent non-covalent complexes (see Fig. 4b), reflects the presence of an additional methylene group in the side chain of l-Glu. In Fig. 5a,b we also show H-bonds around two water molecules. The first of these waters forms four H-bonds and it is part of the “oxy-anion hole”, a motif that is important for stabilization of the negatively-charged tetrahedral intermediate. A unique role of this water molecule, not described before for other hydrolases, was extensively discussed previously^16^. The chemical environment of this water determines directionalities of four H-bonds around it and defines its role as a hydrogen donor to the negatively-charged oxygen atom of the tetrahedral intermediate. The second water molecule highlighted in Fig. 5 forms an H-bond with Thr100(O_γ_) and is placed close to the carbonyl carbon of the AEI. When activated by Lys173 in PGA(wt), this water molecule acts as the nucleophile in the second reaction step. In a catalytically deficient variant of PGA used in these crystallization experiments, the K173M substitution greatly decreases the efficiency of deacylation, allowing visualization of the intermediate state. However, the site occupied by this water becomes accessible only after the first nucleophilic substitution and formation of covalent AEI. Structurally, the active sites of PGA and EcAII are very similar in the presence of covalently bound l-Asp and differences mirror those described for equivalent non-covalent complexes of both enzymes.

The third structure of a covalent assembly, described in this report, represents a complex between PGA(wt) and a citrate anion. The presence of an analogous complex was described by us previously for EcAI(wt), after reinterpretation of earlier data^29^. EcAI is a type I l-asparaginase and is only modestly homologous to either EcAII or PGA. Nevertheless, Fig. 5c demonstrates that covalent complexes of PGA and EcAI with citrate anion are structurally nearly identical.

## Discussion

This report is a continuation of our studies of the catalytic mechanism in l-asparaginases. We have previously shown that hydrolysis by EcAII follows the double-displacement mechanism, described specific roles of the catalytic residues, and presented a plausible model for electron-transfer during catalytic reaction^16^. However, a limitation of the previous work was that it was focused on a single enzyme, thus not allowing generalization of the final conclusions for a broader range of l-asparaginases, especially those with a significant glutaminase activity. In an attempt to mitigate this deficiency, we subjected another l-asparaginase, PGA, to a range of experiments. While PGA has been less extensively studied than EcAII, it is one of only a few l-asparaginases for which basic enzymatic and structural properties have been described^17,18^. PGA shares modest homology with EcAII (48% a.a. sequence identity), but it has vastly different substrate specificity. Whereas EcAII catalyzes hydrolysis of l-Asn with 50–100 times higher efficiency than l-Gln^11^, the latter is the preferred substrate of PGA^17^, hence PGA is referred to as glutaminase-asparaginase. The only other previously characterized glutaminase-asparaginase is an enzyme from *Acinetobacter glutaminasificans* (AGA)^34,39,40^. Moreover, PGA was shown to efficiently hydrolyze other substrates, such as D-Asn and D-Gln, suggesting higher plasticity of its active site when compared to EcAII^17^. Finally, no structure of a productive complex between any l-asparaginase and l-Glu/ l-Gln has been reported to date. Our analysis shows that the structures of such complexes currently present in the PDB^35–37^ do not represent catalytic assemblies.

An additional deficiency of studies of the enzymatic mechanism of asparaginases is related to the fact that all experiments demonstrating formation of covalent AEI, either in the crystals or in solution, involved protonated l-Asp but not the native substrate, l-Asn (or l-Gln). Although we postulated before the basis of this limitation^16^, experimental support was still needed. In this report we present results of high-resolution mass-spectrometry experiments that, in conjunction with structural studies, satisfactorily resolve this problem.

In the first series of experiments employing Single-Quad LC/MS technique, we have shown that two variants of PGA, T100V and K173M, rapidly form covalent AEI's with either l-Asp or l-Glu in a broad pH range. The fast rate of acylation, observed even at neutral pH, must be due to enzymatic activity. These results demonstrate that catalysis by PGA proceeds according to the double-displacement mechanism, as concluded previously for EcAII. Results of structural studies provide a clear description of covalent complexes formed during catalysis and show that AEIs formed by PGA are equivalent to those described for EcAII. Furthermore, these studies demonstrate that Michaelis complexes with l-Asp and l-Glu are structurally equivalent, indicating that, as expected, the mechanism of hydrolysis of both substrates is identical. Therefore, it is reasonable to suggest that the mechanism of catalytic reaction is common to all type II l-asparaginases from mesophilic bacteria, i.e. homo-tetrameric enzymes. A notable additional observation is the mode of binding of l-Glu to the active site pocket. Results obtained here suggest that binding process to the active site of l-asparaginase is more complex in the case of l-Glu (and presumably l-Gln) than that observed for the smaller l-Asp (or l-Asn). Binding of the larger substrate appears to follow two phases, an initial attachment, primarily based on interactions of its α-carboxyl group with the Ser67 (Ser58 in EcAII) and subsequent adjustment of the substrate's molecule in the active site pocket. Complexes with l-Glu described in previous reports^35–37^ represented an assembly resulting from fast attachment, prior to adjustment of the side chain that would create a productive complex. The side chain of l-Glu (or l-Gln) assumes a position and conformation primed for the catalytic process only after such adjustment. This stage is associated with conformational changes of the ASFL necessary to carry out the reaction. Since the size and shape of the active site cavity of PGA is not significantly different from that of EcAII, either composition of the active site or more distant determinants are responsible for the ability to accommodate the larger substrate. This problem, however, was not studied here and requires further investigation.

Additional clues were provided by the high-resolution mass spectrometry (Q-TOF LC/MS) studies of solutions containing PGA(wt), its native substrates (l-Asn and l-Gln) and an external nucleophile (NH_2_OH). These studies were combined with the results of structural studies of an inactive T12V variant of EcAII. Although formation of l-aspartic acid β-hydroxamate (l-AHA) or l-glutamic acid δ-hydroxamate (l-GHA) in these experiments does not immediately prove transient existence of AEIs, the lack of such products in the presence of the EcAII(T12V) does. This conclusion is based on the observation that structures of non-covalent complexes of EcAII(wt) with l-Asp and EcAII(T12V) with l-Asn are nearly indistinguishable, thus formation of AEI is necessary for the reaction with an external nucleophile. The results obtained in these experiments are significant, since they clearly demonstrate the equivalence of enzymatic oxygen-exchange reactions with protonated l-Asp or l-Glu and reactions with the native carboxamides, l-Asn and l-Gln.

One of the structures presented here, acquired somewhat serendipitously, describes a covalent complex between PGA(wt) and a citrate anion. We observed a similar complex^29^ after reinterpreting a previously published structure^41^ of EcAI(wt). Binding of a citrate anion is practically identical in both structures. Interestingly, however, in both cases a covalent complex with citrate is found in a reaction with wild-type enzyme. Since reaction with a citrate anion engages the carboxylate group of the substrate and represents an oxygen-exchange process, it is described by a simple equilibrium (see above). This equilibrium is defined by the ratio of the (forward) acylation and (reverse) deacylation reactions. Most likely, reduction of the reverse reaction rate results in accumulation of covalent AEI in reaction with a citrate anion. This speculation can be easily rationalized. As described earlier, abstraction of H-atom from the primary nucleophile (here Thr20) leads to protonation of an aspartate residue (here Asp101), a process that is critical for high efficiency of the second nucleophilic substitution^16^. An excess of negatively-charged carboxylate groups in citrate anion, compared to canonical substrates, introduces possible “proton-traps”, preventing completion of the critical proton transfer (Thr20 → Asp101). Such transfer may also be restrained by a larger size of the citrate anion compared to a canonical substrate. However, it is significant that the structures of covalent complexes with a citrate anion of two weakly-homologous enzymes (PGA and EcAI) are stereochemically similar to covalent complexes with canonical substrates. This observation suggests that the double-displacement mechanism of catalysis is shared by all tetrameric type II and type I l-asparaginases from mesophilic bacteria. Details of this mechanism were described in our previous report^16^. A general outline is illustrated in the scheme below. The red and blue dots represent oxygen- and nitrogen-based groups, respectively, while l-ASNase, S, TI, and AEI stand for l-asparaginase, substrate, tetrahedral intermediate, and covalent acyl-enzyme complex, respectively. This scheme emphasizes that Thr residue acts as the primary nucleophile and the second nucleophilic attack is initiated by the active site water molecule. In reactions with canonical substrates, l-Asn and l-Gln, one step of the catalytic reaction, associated with departure of ammonia molecule, is irreversible.
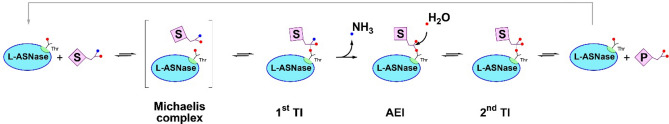


Due to topological and structural similarity of the active sites, it is tempting to suggest that this mechanism is shared by other asparaginases, such as those from extremophilic bacteria or eukarya. This suggestion, however, requires additional verification.

## Materials and methods

### Reagents and sequencing services

The following reagents: l-Asn (Catalog No. 51363), l-Asp (Catalog No. 11189), l-Gln (Catalog No. G3126), l-Glu (Catalog No. G1251), D-Glu (Catalog No. G1001), sodium carbonate (Catalog No. 106395), sodium cacodylate trihydrate (Catalog No. 20840), sodium citrate dihydrate (Catalog No. W302600), MES buffer sodium salt (Catalog No. RES011M-A7), HEPES buffer (Catalog No. H3375), l-aspartic acid β-hydroxamate (l-AHA, Catalog No. A6508), isopropyl β-D-1-thiogalactopyranoside (IPTG, Catalog No. PHG0010), glutaraldehyde (25% in H_2_O, Catalog No. G5882), trichloroacetic acid (Catalog No. T9159), and 8-hydroxyquinoline (Catalog No. H6878) were procured from Millipore-Sigma. All crystallization screens used in preliminary experiments were purchased from Hampton Research.

The expression vector pET22b( +) was purchased from Millipore-Sigma (Catalog No. 69744-3). The BL21 (DE3) RIPL expression cells (Catalog No. 230280) and PfuUltra II Hotstart PCR Master Mix for QuikChange reactions (Catalog No. 600850) were obtained from Agilent Technologies. The Nessler's reagent was prepared according to the protocol available at https://sites.chem.colostate.edu/diverdi/all_courses/CRC%20reference%20data/special%20analytical%20reagents.pdf. All other chemicals used were purchased from different vendors at the highest available grade.

DNA sequencing was performed by Macrogen Corp., using T7-promoter and T7-terminator universal primers. Additional reagents, tools, and kits used in cloning, extraction, and purification steps are identified along with description of specific procedures.

All buffers were prepared fresh and used within two weeks. The Nessler’s reagent was used within two months of its preparation. The oxin/carbonate reagent was always prepared fresh prior to the assay. If not indicated otherwise, all other reagents or kits were prepared and used according to the manufacturer recommendations.

### Preparation of the PGA samples

The sequence of PGA was extracted by PCR from genomic DNA, ATCC 47054. The PGA gene with N-terminal (His)_6_ sequence was generated by two PCR steps, first with the forward primer 5′- cat cat cat cat cac AAA GAA GCC GAA ACC CAA CAG AAG C-3′ and the reverse primer 5′-AAA AAA ctc gag tta GTA CTC CCA GAA GAT CCG CTG CAG C-3′ and then with the forward primer 5′-AAA AAA cca tgg gc agc agc cat cat cat cat cat cac AAA GAA GCC GAA ACC C-3′ and the same reverse primer. The PGA fragment was excised by NcoI and XhoI and cloned into pET22b( +). However, levels of PGA secretion, controlled by the pelB leader in the pET22b( +) vector, were disappointing. Therefore, we subsequently excised the sequence encoding the pelB leader by a QuikChange procedure with the forward primer 5′-GAA GGA GAT ATA CAT ATG ggc agc agc cat cat cat cat cat cac-3′ and the reverse primer 5′-gtg atg atg atg atg atg gct gct gcc CAT ATG TAT ATC TCC TTC-3′. Subsequently, the TEV protease cleavage site was inserted, following the (His)_6_-tag, by a QuikChange procedure with the forward primer 5′-c cat cat cat cat cat cac GAGAACCTGTACTTCCAGGGT aaa gaa gcc gaa acc caa c-3′ and the reverse primer 5′-g ttg ggt ttc ggc ttc ttt ACCCTGGAAGTACAGGTTCTC gtg atg atg atg atg atg g-3′. The resulting vector is referred to as pZD312. The vector encoding the PGA(T100V) mutant (referred to as pZD313) was generated by a QuikChange procedure using pZD312, the forward primer 5′-TCGTCATCACCCACGGCGTCGATACCCTCGAAGAAACC-3′, and the reverse primer 5′-TTCTTCGAGGGTATCGACGCCGTGGGTGATGACG-3′. The PGA(K173M) mutant was generated with use of the forward primer 5′-GCGATGTGAGCATGGCGGTCAACATCAAGACCG-3′ and the reverse primer 5′-TTGATGTTGACCGCCATGCTCACATCGCGGCCGG-3′.

For expression, an appropriate vector was transformed into BL21(DE3) RIPL cells. After induction with IPTG (c_final_ = 0.5 μM), expressions were carried out overnight (16–18 h) at 18 °C in the presence of ampicillin and chloramphenicol. The cell pellet was resuspended in 50 mM Tris, 0.5 M NaCl, pH 8.0 and lysed by microfluidizer. The lysate was cleared by centrifugation at 15,000 rpm at 4 °C for 30 min and filtration through the 0.45 μm filter. Initially, the protein was purified using a HisTrap HP 5 ml column (GE Healthcare), according to manufacturer’s recommendations. Subsequently, sample was dialyzed overnight at 4 °C against a buffer containing 50 mM Tris pH 8, 200 mM NaCl and the TEV protease, to cleave the N-terminal (His)_6_-tag. Sample was applied again on the HisTrap HP 5 ml column and the flow-through was collected. Finally, after concentrating, protein was subjected to size-exclusion purification using the Superdex 200 16/60 prep grade column (GE Healthcare) with 50 mM HEPES, 200 mM NaCl, pH 7. Solution of purified protein was concentrated to 15 mg/ml and used immediately for crystallization experiments or frozen at -80 °C for future use.

### LC/MS analysis of the AEI formation

Mass spectrometry data were acquired on an Agilent 6100 Series Quadrupole LC/MS System (Agilent Technologies, Inc., Santa Clara, CA) equipped with an electrospray source, operated in the positive-ion mode. Separation was performed on Zorbax 300SB-C3 Poroshell column (2.1 mm × 75 mm; particle size 5 μm). Mass spectra were recorded across the range 300–2000 m/z. The UV signal was collected at 280 nm with a reference at 360 nm. Data acquisition and analysis were performed using OpenLAB CDS ChemStation Edition C.01.05. Solutions containing 50 mM buffer (sodium acetate, pH 4–5.4; sodium cacodylate, pH 5.6–7.0; HEPES, 7.2–8.0), 3 μM PGA(K173M) or 3 PGA(T100V), and 2.5 mM l-Asp or l-Glu were prepared immediately prior to MS analysis.

### High-resolution LC/MS analysis of the l-Asn (l-Gln) hydrolysis by PGA and EcAII in the presence of external nucleophile (NH_2_OH)

Mass spectrometry data were acquired on an Agilent 6520 Accurate-Mass Q-TOF LC/MS System, (Agilent Technologies, Inc., Santa Clara, CA) equipped with a dual electro-spray source, operated in the positive-ion mode. Separation was performed on Poroshell 300SB-C18 column (2.1 mm × 75 mm; particle size 5 μm). The analytes were eluted first at a flow rate of 0.5 ml/min with 0 to 20% acetonitrile gradient over 3 min, then at a flow rate of 1 ml/min with 20 to 100% acetonitrile gradient over the next 3 min and holding the organic solvent for 1 min, where 0.1% aqueous formic acid was exchanged for 0.1% formic acid in acetonitrile. The instrument was used in full-scan TOF mode. MS source parameters were set with a capillary voltage of 4 kV, the fragmentor voltage of 100 V and skimmer 65 V. The gas temperature was 350 °C, drying gas flow 12 l/min and nebulizer pressure 55 psig. Data were acquired at high resolution (3200 m*/z*), 4 GHz. To maintain mass accuracy during the run time, an internal mass calibration sample was infused continuously during the LC/MS runs. Data acquisition and analysis were performed using MassHunter Workstation Data Softwares, LCMS Data Acquisition (version B.06.01) and Qualitative Analysis (version B.07.00). For quantitative analysis the mass spectrometry response of ions was measured from the area under chromatography peak of Extracted Ion Chromatogram (EIC).

The high-resolution LC/MS experiments can be divided into three groups. In all cases solutions were buffered by 25 mM HEPES pH 7.7. Measurements within the first group aimed at the determination of experimental mass-to-charge (m/z) values for all expected low-molecular-weight components, here l-Asn, l-Asp, l-Gln, l-Glu, l-AHA, l-GHA, as well as HEPES molecule. In this case analytes were dissolved at concentrations between 2 μM and 20 mM (the results are shown in the Supplementary Information). In the second (main) group, catalytic reactions were monitored for solutions containing the enzyme (PGA, EcAII or EcAII(T12V) at concentration 27.8 nM), the substrate (l-Asn, l-Gln or l-Asp at concentration 8 mM) and the external nucleophile, NH_2_OH (conc. 0.4 M). The first data acquisition was at 10 min after beginning of a reaction and subsequent measurements were acquired at 30 min intervals over total 480 min (see also Supplementary Information). The third group of experiments aimed at establishing relations between the equipment response (reported as areas under the extracted ion chromatogram peaks) and pre-defined concentrations of studied component. This information was crucial for determining concentrations of critical components throughout the course of catalytic reaction.

### Crystallization and collection of X-ray data

In all experiments, protein solutions at a concentration 12.5–13.5 mg/ml in 50 mM HEPES buffer (pH 7.0) and 200 mM NaCl were used. All crystallizations were conducted in the hanging drop format, with droplets obtained by mixing equal volumes of protein and precipitant solutions. After short soaking in appropriate cryo solutions (see Table 1), crystals were rapidly frozen in a stream of nitrogen cooled to 100 K. Diffraction data were collected at the Advanced Photon Source, Argonne National Laboratory (Argonne, IL, USA), on beamline 22-ID (crystals 1 through 6) or beamline 22-BM (crystal 7). Images were processed and scaled with HKL3000^42^. Details of data collection and the processing statistics are presented in Table 2.

### Structure solution and refinement

All structures were solved independently by molecular replacement using the program Phaser^43^ in order to minimize possible bias. Isomorphous structures were ultimately brought to the same sections of the unit cell. We used protomer A of PDB entry 4pga^19^ as the search model, after removing the ligand, solvent molecules, and the HR and ASFL sections (residues 18–39). In all cases easily identifiable molecular replacement solutions were subjected to rigid-body refinement at the resolution of 2.5 Å with the program Refmac5^44^, followed by several cycles of refinement of positions and isotropic atomic displacement parameters (Bf’s) for non-H atoms with aid of the same program. In subsequent rounds of crystallographic refinement the resolution was gradually extended to reach the limits of experimental data. In the final stages of refinement, an anisotropic approximation of atomic displacement factors was implemented for structures 2, 3, and 6. Models were continuously inspected using the program Coot^45^ and appropriate corrections were introduced, including proper modeling of residue 173 and the ordered sections of ASFL. Ligand and solvent molecules were gradually incorporated in the structure based on difference electron density peaks. The near-final models were evaluated by the MolProbity server^46^, subjected to a subsequent refinement. The statistics for the final structural models are shown in Table 2.

### Measurements of enzyme kinetics

Measurements of absorption were performed with the NanoDrop One (Thermo Sci.). For kinetic studies we used two previously described assays^27^. The aim of these studies was the determination of the *k*_cat_ values under conditions of steady-state kinetics, without estimating the associated K_M_ parameters (see “Results”). Hydrolysis of l-Asn and l-Gln was examined by monitoring formation of ammonia with Nessler’s reagent. Reactions were conducted in 50 mM MES buffer (pH 6) with defined concentrations of the enzyme and l-Asn or l-Gln (see Supplementary Fig. S1 a-c) and four different substrate concentrations, varying between 20 and 83 mM. At predetermined intervals, 40 μl aliquots were withdrawn and the reaction was rapidly quenched by addition of 40 μl 12% (w/v) solution of trichloroacetic acid. After pelleting any precipitate by centrifugation, 30 μl of the supernatant was mixed with 30 μl of the Nessler's reagent and absorption at 480 nm (A_480_) was recorded immediately. Measured A_480_ values were converted to concentrations of ammonia using published value of the absorption coefficient of the Nessler's product ε_480_ = 1115 ± 40 M^−1^·cm^−1^. Since the results obtained for different substrate concentrations were identical within 3%, those obtained for the highest substrate concentration (83 mM) are reported. Progress curves, shown in Supplementary Fig. S1 a-c, represent averages from four independent measurements.

The activity of the PGA(K173M) mutant was assessed by monitoring hydrolysis of the l-asparagine analog l-aspartic acid β-hydroxamate (l-AHA) as the substrate at pH 5 (50 mM MES). Release of hydroxylamine was monitored via reaction with 8-hydroxyquinoline at basic pH, which leads to formation of green oxindole dye (ε_705_ = 1.75·10^4^ M^−1^·cm^−1^,^47^). Details of this assay were described previously^16,27^. This assay was also conducted at four different concentrations (2, 3.5, 5, and 7.5 mM) of l-AHA, and the resulting *k*_cat_ values were indistinguishable. In this case, we also report values of *k*_cat_ for the highest substrate concentration. In all experiments, we used reference ("blank") solutions; their absorptions were subtracted from the actual measurements. The blanks had compositions identical to the reaction mixtures but did not include substrates. Similar to the previous assay, the progress curve (see Supplementary Fig. S1d) represents averages from four independent measurements.

## Supplementary information

Supplementary file1

## Data Availability

Coordinates and structure factors of the models described in this manuscript were deposited in the Protein Data Bank under accession numbers 6wyw, 6wyx, 6wyy, 6wyz, 6wz4, 6wz6, and 6wz8.
