# Supplementary material for: Generalized enzymatic mechanism of catalysis by tetrameric l-asparaginases from mesophilic bacteria
Source: Sci Rep. 2020 Oct 15;10:17516. doi: 10.1038/s41598-020-74480-4 (PMC7567106; doi:10.1038/s41598-020-74480-4)
Supplement: Supplementary file 1 — Supplementary file1 [file 41598_2020_74480_MOESM1_ESM.pdf]

Supplementary Information

Generalized Enzymatic Mechanism of Catalysis by Tetrameric L-Asparaginases from Mesophilic Bacteria

Pawel Strzelczyk[1,a], Di Zhang[1,a], Marzena Dyba[2], Alexander Wlodawer[1] and Jacek Lubkowski[1*]

[1]Macromolecular Crystallography Laboratory, National Cancer Institute, Frederick, Maryland

[2]Basic Science Program, Structural Biophysics Laboratory, Frederick National Laboratory for Cancer Research sponsored by the National Cancer Institute, Frederick, MD

Section 1. Examples of progress curves acquired during reactions of PGA and two of its variants with L-Asn, L-Gln, and L-AHA

Section 2. Raw data from LC/MS studies conducted with the Quadrupole LC/MS System

Section 3. Raw data from high-resolution LC/MS studies conducted with the Q-TOF LC/MS System

Section 1. Examples of progress curves acquired during reactions of PGA and two of its variants with L-Asn, L-Gln, and L-AHA

Plots illustrated below were generated based on results of assays described in the main manuscript.

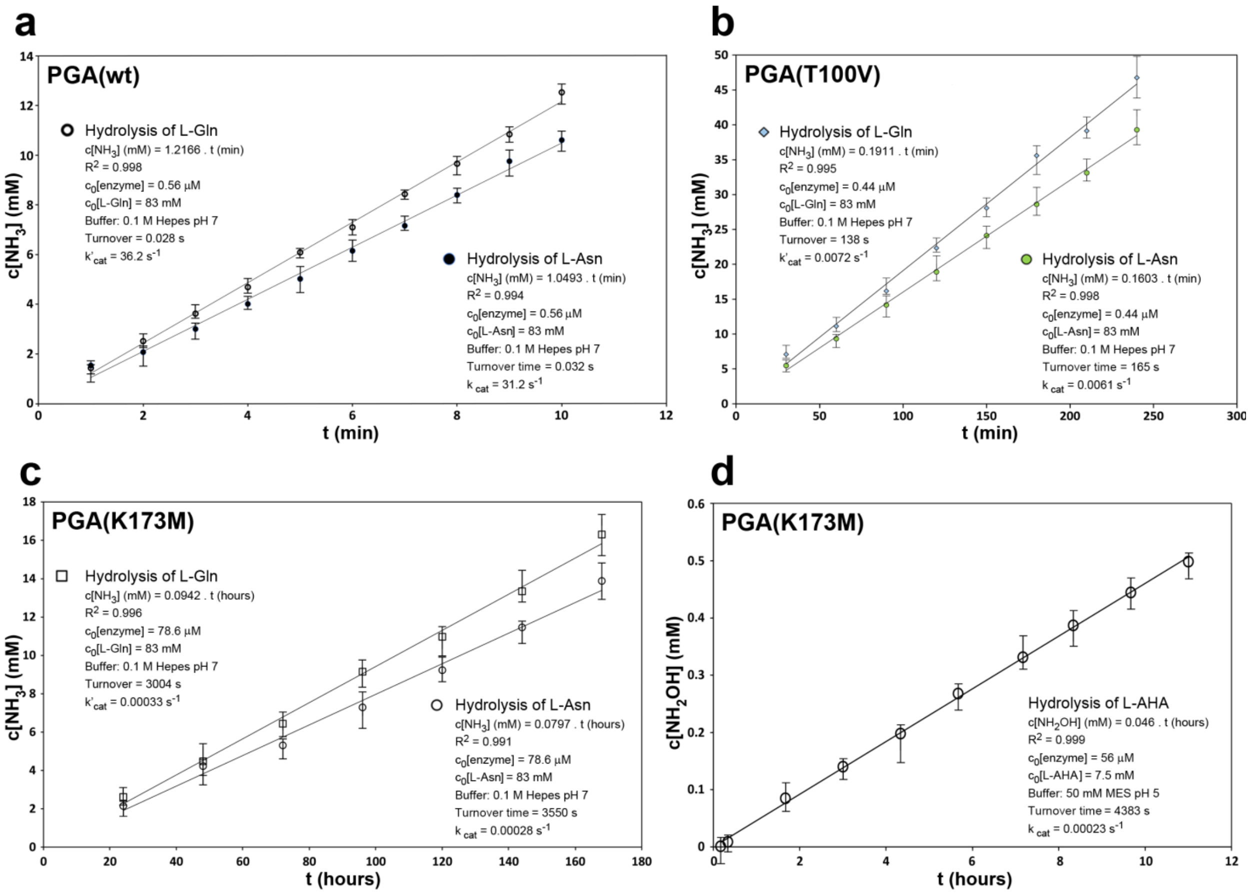

Figure S1. Kinetic studies PGA and its mutants. Panels **a-c** describe hydrolysis of L-Asn and L-Gln by PGA(wt), PGA(T100V), and PGA(K173M), respectively. Progress of hydrolysis was measured by an increase of the product (NH_3) concentration. Hydrolysis of L-aspartic acid β-hydroxamate (L-AHA) by PGA(K173M), monitored by measurements of an increase of NH_2OH concentration, is illustrated in the panel **d**. The initial conditions (concentrations of reactants and pH) are shown within each panel, while details of assays are described in the Materials and Methods in the main manuscript. Each data point represents an average of four independent experiments and the spreads of individual measurements are illustrated by vertical error bars. Under conditions of the assays progress curves are linear and the least squares approximations, with the agreement reflected the Pearson product coefficient (R^2), are shown in each panel. Also, resulting values of turnover times per single active site and their inverses (~k_{cat}) are shown in each panel.

Section 2. Raw data from LC/MS studies conducted with the Quadrupole LC/MS System

Below are two examples of raw data acquired during LC/MS measurements of PGA(T100V) and L-Asp mixtures at two different pH values. Similar data were obtained for mixtures with L-Glu, as well as solutions containing PGA(K173M) instead of PGA(T100V), as described in Materials and Methods in the main manuscript. Each measurement was conducted in triplicate and the average value represented one point on the graph shown in Fig. 1a.

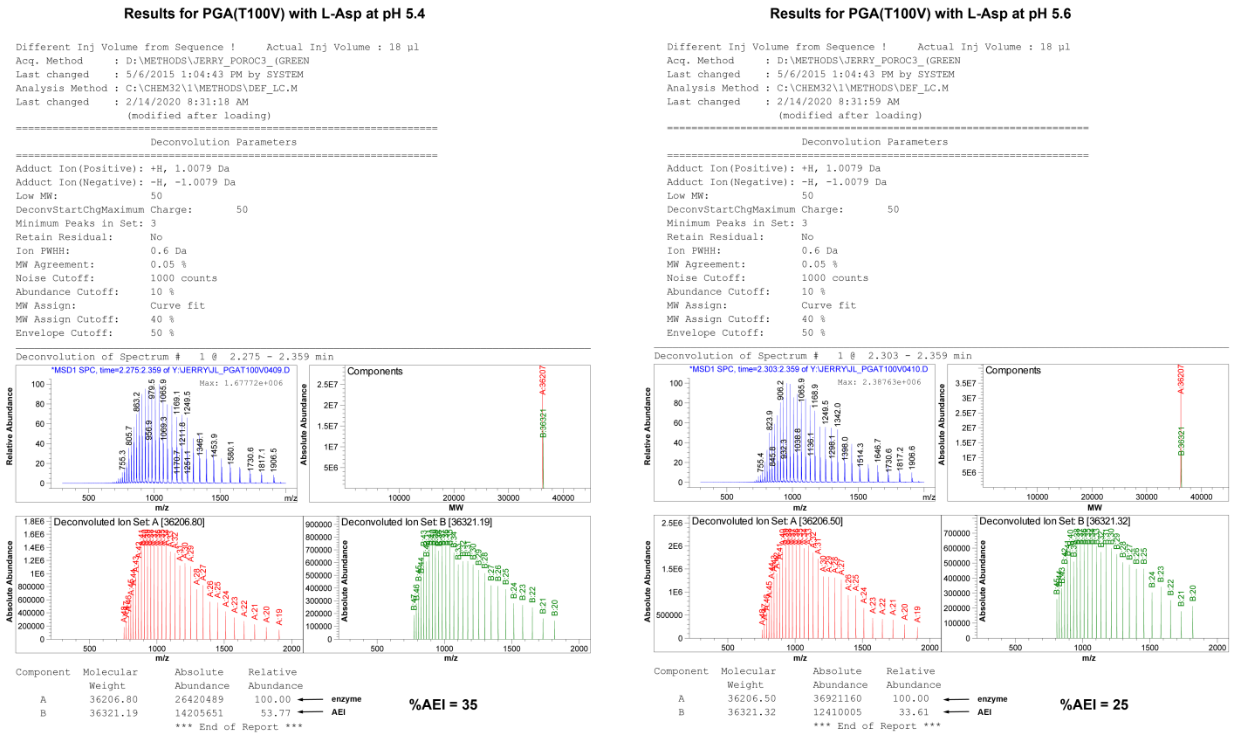

Figure S2. The raw data acquired on an Agilent 6100 Series Quadrupole LC/MS System (Agilent Technologies, Inc., Santa Clara, CA) equipped with an electrospray source, operated in the positive-ion mode. Results are shown for two solutions containing PGA(T100V) mutant with L-Asp and differing by pH, as shown at the top of the figure. The % content of AEI is calculated directly from the relative abundancies (indicated by arrows) of components (PGA mutant and AEI), according to the following formula: **% AEI** = abundance of AEI [%]) /{(abundance of AEI [%]) + (abundance of EcAII(T100V) [%]).

Section 3. Raw data from high-resolution LC/MS studies conducted with the Q-TOF LC/MS System

As discussed in the main manuscript, values of mass-to-charge (m/z) were initially determined for all low-molecular-weight components. Below is shown an example of the mass spectrum acquired for L-Asn.

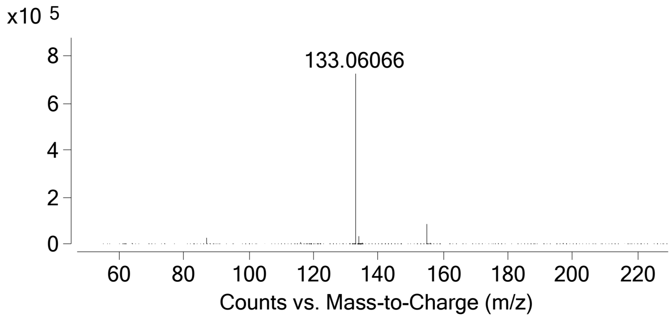

A complete list of m/z values acquired during these measurements is presented in the Table below.

**Compound**	**m/z**	
	**Measured**	**Calculated**
L-Asn	133.06066	133.06076
L-Asp	134.04451	134.04478
L-AHA	149.05607	149.05568
L-Gln	147.07667	147.07641
L-Glu	148.06008	148.06043
L-GHA	163.07120	163.07133
HEPES	239.10536	239.10600

Subsequently, a series of high-resolution LC/MS measurements were conducted for mixtures containing the enzyme (PGA(wt), EcAII(wt) or EcAII(T12V) at the concentration of 27.8 nM, substrate (L-Asn or L-Gln) at the concentration 8 mM, and external nucleophile NH_2OH at the concentration 0.4 M, in 24 mM buffer (Hepes pH 7.7). Figure shown below illustrates **extracted ion chromatograms (EIC)** for monitored ions **a)** 133.06 m/z, L-Asn, **b)** 134.04 m/z, L-Asp, and **c)** 149.06 m/z, L-AHA at different reaction times. The first chromatogram, illustrated at the top of each panel in the Figure below, was acquired 10 minutes after a reaction has started. Subsequent chromatograms, shown gradually downward, were acquired at 30 min intervals over the 480 min total time of experiment. To quantify each of L-Asn, L-Asp, and L-AHA at any given reaction time, an area under the peak of EIC was integrated. Finally, the recoded areas were converted to concentrations using appropriate calibration curves. The latter were established by a series of high-resolution LC/MS experiments with solutions containing the studied components at known concentrations in the same buffer as used for reactions. For each solution, an area under the peak of EIC could be determined and the relation between area and concentration was determined for each component, L-Asn, L-Asp, L-AHA (shown here), as well as L-Gln, L-Glu and L-GHA.

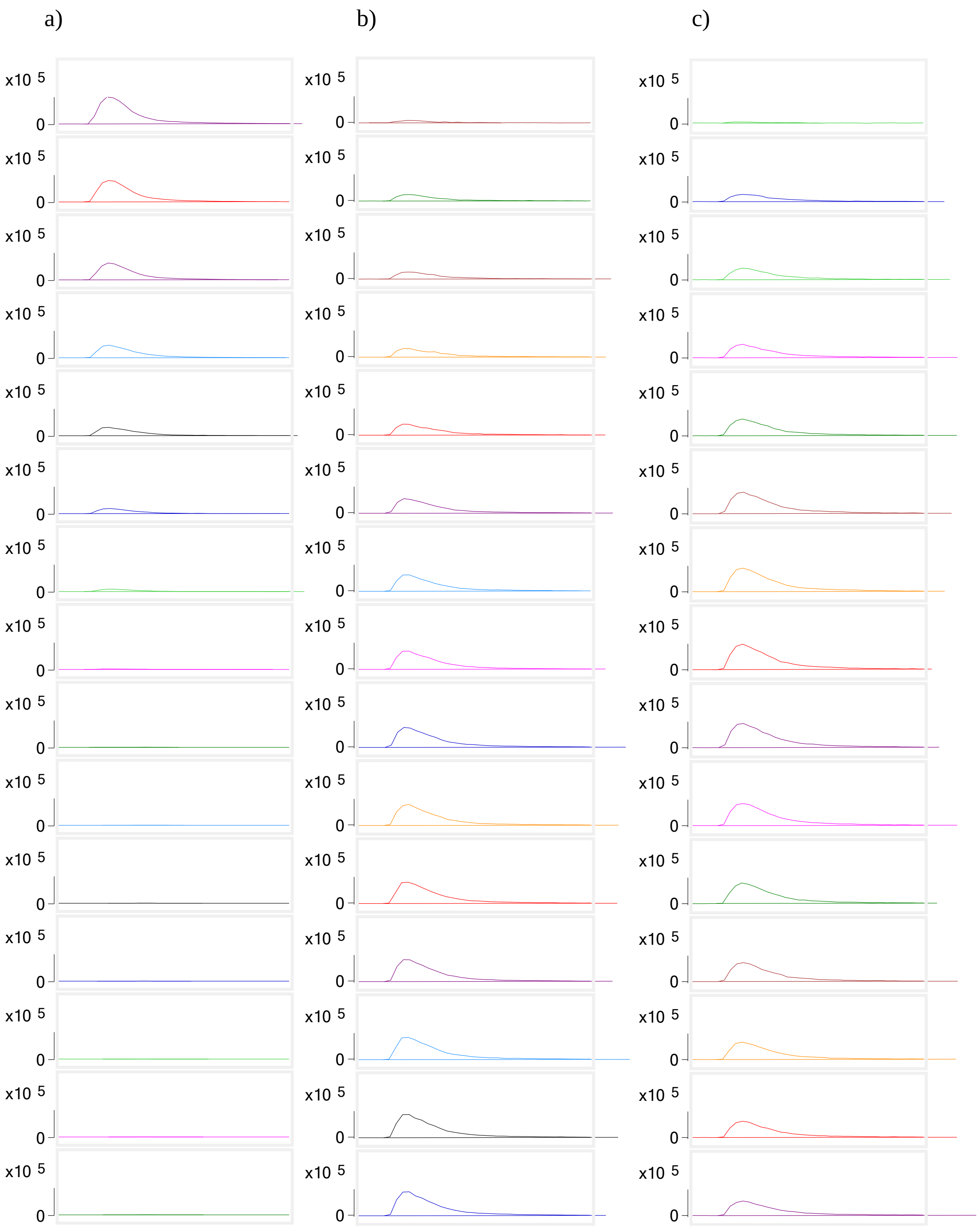
a)
b)
c)
x10 5
0
